# Investigation of the Fracture Behaviour of Al–CFRP Cross-Lap Joint Fabricated by Coaxial One-Side Resistance Spot Welding

**DOI:** 10.3390/polym16060738

**Published:** 2024-03-08

**Authors:** Sendong Ren, Hao Chen, Ninshu Ma, Jingjia Chen, Shuhei Saeki, Yoshiaki Iwamoto, Jianguo Yang

**Affiliations:** 1 College of Mechanical Engineering, Zhejiang University of Technology, Hangzhou 310014, China; chenhao112700@outlook.com (H.C.); yangjg@zjut.edu.cn (J.Y.); 2 Joining and Welding Research Institute, Osaka University, Osaka 567-0047, Japan; ma.ninshu.jwri@osaka-u.ac.jp (N.M.); jingjia.chen@jwri.osaka-u.ac.jp (J.C.); 3 Dengensha Toa Co., Ltd., Kanagawa 214-8588, Japan; saeki.s@dengenshatoa.co.jp (S.S.); iwamoto.y@dengenshatoa.co.jp (Y.I.)

**Keywords:** coaxial one-side resistance welding, CFRP, cross-lap joint, multi-scale characterization, fracture behaviour

## Abstract

In the present research, coaxial one-side resistance spot welding was performed to join Al5052 and CFRP sheets with different welding currents. The mechanical performance of the cross-lap joint was clarified experimentally. The cross-section of the welded joint and the fracture surfaces was subjected to multi-scale characterization. The fracture behaviours and mechanisms of cross-lap joints are discussed in detail. The results showed that the thermal degradation of CFRP was detected on the cross-section under a 6000 A welding current and the O element was enriched in the decomposed area. The joining zone could be divided into four subregions according to their morphology, which were defined, from outside to inside, as the squeezed zone, the adhesion zone, the cohesion zone and the decomposed zone. After welding, the O-C=O bond disappeared on the CFRP surface while the O=C-N bond was detected on the Al5052 surface. The cross-lap joints demonstrated brittle and ductile fracture behaviours in a cross-tension test, which included two sub-modes: brittle-transition mode and ductile-degradation mode. The transformation of failure modes had a relationship with the heat input and corresponding joining zone composition. The maximum cross-tension load was about 1279 ± 40 N with a welding current of 5600 A.

## 1. Introduction

The ongoing requirement for lightweight design in the automobile industry promotes the development and application of new-generation lightweight structural materials. Compared with traditional metals, carbon fibre reinforced plastic (CFRP) demonstrates some excellent performances which are desired by automobile manufacturers: excellent strength-to-weight ratio, low density, outstanding abrasion resistance and superior energy absorption capacity, as well as great flexibility in the design of structure and functions [1,2]. Currently, the main application of CFRP in mass-market products is in metal–CFRP hybrid structures, which offers a surprising potentiality for weight reduction and gives consideration to both strength and economy [3], while the rapidly increasing necessity of production capacity and quality standards also presents a significant challenge to the technology of joining dissimilar materials.

Automotive manufacturers have accepted two basic techniques to join metal and CFRP sheets in lightweight body-in-white designs, which are mechanical fastening and adhesive bonding [4]. These are both suitable for joining dissimilar materials with significant differences, particularly for the thin-wall structures. With the development in technology, some thermal-based joining technologies have been proposed to connect metal and CFRP. Thermal-based joining aims to melt partially plastic at the metal–CFRP interface and form chemical bonds or micro-mechanical interlocking to provide cohesion, thereby eliminating the extra weight induced by the fastener and the adhesive [5]. The mechanical performance of metal–CFRP joints is the most critical factor in evaluating the quality and reliability of the thermal-based joining technology.

Many scholars have conducted relevant research into the mechanical performance of metal–CFRP joints prepared by different thermal-based joining approaches. He et al. [6] studied the effect of linear heat input on the mechanical properties of DP780–CFRP laser welding. The shear force reached a peak of about 1959.4 N at 50 J/mm. Jeong-Won Choi et al. [7] connected pure Ti and CFRP via friction stir welding. An interface temperature between the melting point and decomposition point of CFRP was of benefit in forming superior tensile shear strength. In addition, Geng et al. [8] reported experimental and numerical research of friction spot joining between Al6061 and CFRP. The maximum shear strength was about 5.96 MPa. Staab et al. [9] investigated the local shear strength distribution of Al–CFRP ultrasonic torsion welding. They found that the local tensile shear strength was a function of the joining area. Guo et al. [10] joined CFRP and 5083 aluminium alloy by induction brazing. The tensile strength was 176.5 MPa and the fatigue limit was 71.68 MPa. Yao et al. [11] investigated the fatigue behaviour of Al–CFRP spot-welded joints by electromagnetic pulse welding. The tensile strength reached about 7.49 kN, which was about 92.24% that of the aluminium. In previous research by the authors, coaxial one-side resistance spot welding (COS-RSW) was proposed to connect Al5052 and CFRP, and the joining strength of a single lap joint could reach about 20 MPa [12].

Different research works have clarified the fracture mechanism and modes in metal–CFRP thermal-based joints. Xia et al. [13] studied the fracture behaviours of DP590–CFRP laser-welded joints. Three distinct fracture modes were observed under different laser powers: interface failure occurred at low power, cohesion failure happened at medium power and the mixed mode appeared at enormous power. Dong et al. [14] investigated the friction stir spot welding between Al5052 and CF-PEEK. The interface could be divided into four parts. The adhesive fracture, cohesive failure and the mixed fracture mode occurred in different zones. Staab and Balle [15] employed ultrasonic torsion welding to join AA5024 and GF/CF-PEEK. Both the adhesive bond and the cohesive bond were detected as the joining mechanism. For the COS-RSW between Al and CFRP, the failure mode changed from brittle to ductile with the increased welding current [16]. Generally, the fracture mechanism and different modes had a close correlation with the local bonding strength distribution, which was significantly affected by the interface temperature. The melting of CFRP was necessary to form bonding and the thermal degradation had a negative influence on joining strength.

All the research works mentioned above focused on the single lap joint and the corresponding tensile shear strength. In addition, the cross-tension strength is also necessary to evaluate the reliability of metal–CFRP joining. Ota et al. [17] compared the tensile shear strength and cross-tension strength of Al–CFRP friction stir spot-welded joints. The values were 10.2 kN and 1.92 kN, respectively. Rana et al. [18] clarified the mechanical performance of Al–HDPE–Al sandwich sheets fabricated by friction stir spot welding. The lap-shear test, cross-tension test and peel test were performed. The failure load in the peel test was relatively lower than others. Due to the different loading directions, the fracture process and macro strength of the cross-lap joint should have had an apparent discrepancy with single-lap joint [19]. However, the reports of cross-tension tests on metal–CFRP thermal-based joining were insufficient and focused on friction welding. There is still a research gap in other welding approaches, especially coaxial one-side resistance welding.

In the present research, the cross-lap joint of Al5052 and CFRP dissimilar materials was prepared via coaxial one-side resistance welding. The cross-tension test was processed to evaluate the mechanical performance of COS-RSW joints with different welding parameters. The cross-section and fracture surfaces of joints were subjected to multi-scale characterizations to clarify the detailed fracture mechanism under distinct welding conditions. The joining zone was divided into subregions according to the surface morphology on the welded sheet. The specific fracture behaviours and mechanisms are also discussed in detail, which provides a reference to understanding COS-RSW and improving the joining quality.

## 2. Experimental Methods

### 2.1. Materials

**Figure 1:**
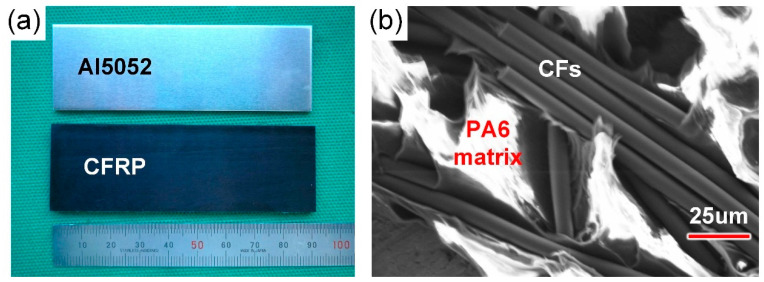
(**a**) The prepared welded sheets and (**b**) microstructure of CFRP.

The bonded materials in the present study were Al5052-O (Northeast Light Alloy, Harbin, China) and CFRP (Genius Advanced Material, Hefei, China.). The Al5052 sheet was machined into a dimension of 90 × 30 × 2 mm. Then, the silane coupling agent OFS-6020 (Dow Corning, San Francisco, CA, USA) was employed to pretreat the Al surface to improve the connection capability between inorganic (Al5052) and organic (CFRP) substances. The components of CFRP were 80 wt% PA6 and 20 wt% short-cut carbon fibres (CFs). The PA6 and CFs were mixed and extrusion moulded into 90 × 30 × 2 mm sheets. The prepared welded sheets and the microstructure of CFRP are illustrated in Figure 1. In previous research by the authors, the melting temperature and decomposition temperature of PA6-CFRP were gauged as about 220 °C and 340 °C, respectively [20].

### 2.2. COS-RSW Process and Parameter

**Figure 2:**
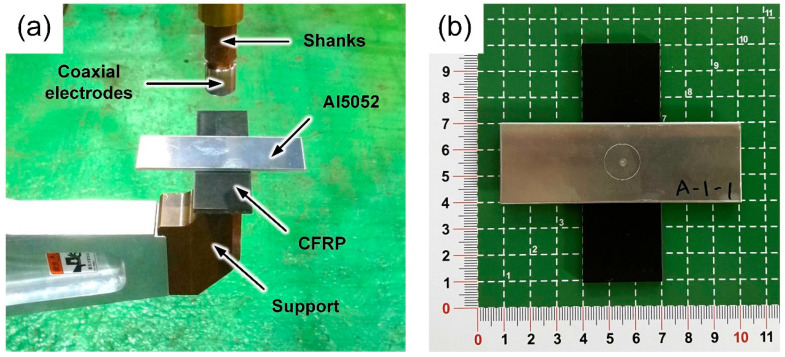
(**a**) The actual welding setup and (**b**) COS-RSW joint.

COS-RSW was proposed by Dengensha TOA company, who used a single-sided electrode couple to create a current loop with metal sheet, thereby overcoming the problem of traditional resistance spot welding without joining insulators like plastic. The principle of COS-RSW was introduced clearly in the previous study [21]. Figure 2 shows the actual welding setup and welded joint. The Al5052 sheet and CFRP sheet were cross-overlapped on the support. The coaxial electrode couple was fixed on the shanks to apply the welding current, electrode force and water cooling condition. In this study, the influence of welding current is discussed since it had a significant effect on Joule heat generation. The welding time and electrode force were unchanged, as summarized in Table 1.

### 2.3. Cross-Tension Test

**Figure 3:**
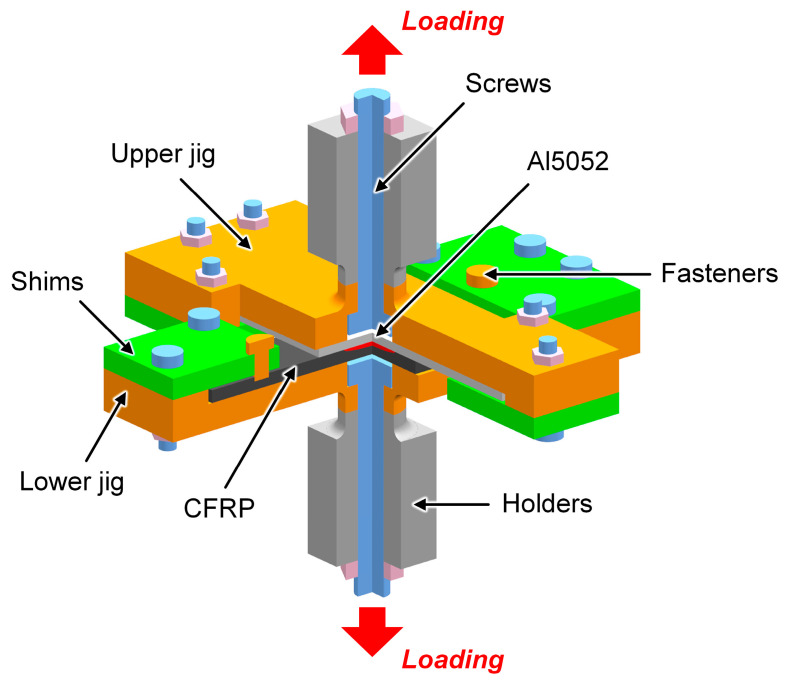
The illustration of cross-tension test.

The mechanical performance of the COS-RSW joint was evaluated via the cross-tension test, as depicted in Figure 3. The experiment was performed on an LD24.204 universal testing machine (Lishi Instrument, Shanghai, China) at a room temperature of about 25 °C. The crosshead speed was about 1 mm/min. Three samples were measured for each case to calculate the average and standard deviation of load. It should be mentioned that the overall dimension of the COS-RSW joint was different from the standard [22], while its proportions were kept the same.

### 2.4. Multi-Scale Characterizations

The COS-RSW joint was cut at the center of the overlap zone along the CFRP direction to obtain the cross-section. It was then subjected to cold mounting and a standard polish procedure. The macro profile of the molten zone was imaged on a Nikon SMZ-745T (Nikon, Tokyo, Japan) stereo microscope. The finer observation was performed on a Phenom XL (Nanoscience Instruments, Phoenix, AZ, USA) scanning electron microscope (SEM) with an acceleration voltage of 10 kV. The energy dispersive X-ray spectrometry (EDS) analysis was processed on an Amptek X-123 Fast SDD (Amptek, Bedford, MA, USA) to clarify the elements’ distribution.

After the cross-tension test, the fracture surface was analyzed on a Keyence VR-5000 (Keyence, Itasca, IL, USA) digital microscope to measure the relative height distribution on the CFRP sheet and characterize the macro-morphology of the joining zone. The microstructure of CFRP in the subregions of fracture surface, especially the residual plastic on the Al5052 sheet, was clarified by the SEM observation. X-ray photoelectron spectroscopy (XPS) was performed based on a Thermofisher Nexsa (Thermo Fisher Scientific, Waltham, MA, USA) with an Al-Kα X-ray source to analyse the chemical bond information in the joining zone.

## 3. Results and Discussion

### 3.1. Analysis of Joining Zone on Cross-Section

**Figure 4:**
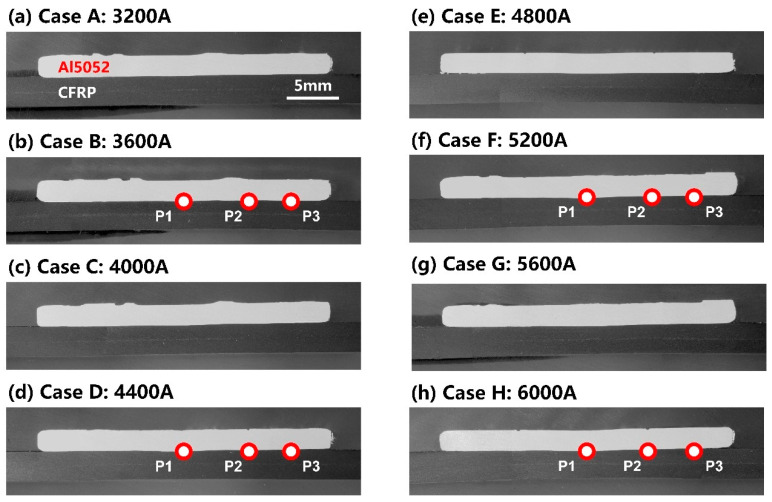
The macro profile of the cross-sections in each welding condition and three typical positions for microscope observation.

The macro profile of the cross-section is shown in Figure 4. The Al5052 sheet and CFRP sheet presented bright and dark colours, respectively. Differently from the metal, the boundary of the molten zone in the CFRP sheet was indistinguishable. Since the plastic was a noncrystal material, the melting and re-solidification process would not introduce differences to its microstructure unless the plastic was decomposed to generate microbubbles. However, the polymer matrix was continuous in the macro-observation. It showed that the thermal degradation of CFRP was not obvious in the present welding conditions. In the center of overlap zone, the Al was slightly pressed into the CFRP, which indicated that the molten plastic was squeezed out from its original location. Since the interface temperature always increased with a larger welding current and decreased sequentially from the inside out, the microstructures of P1, P2 and P3 in the four welding conditions were subsequently compared via microscope observation.

**Figure 5:**
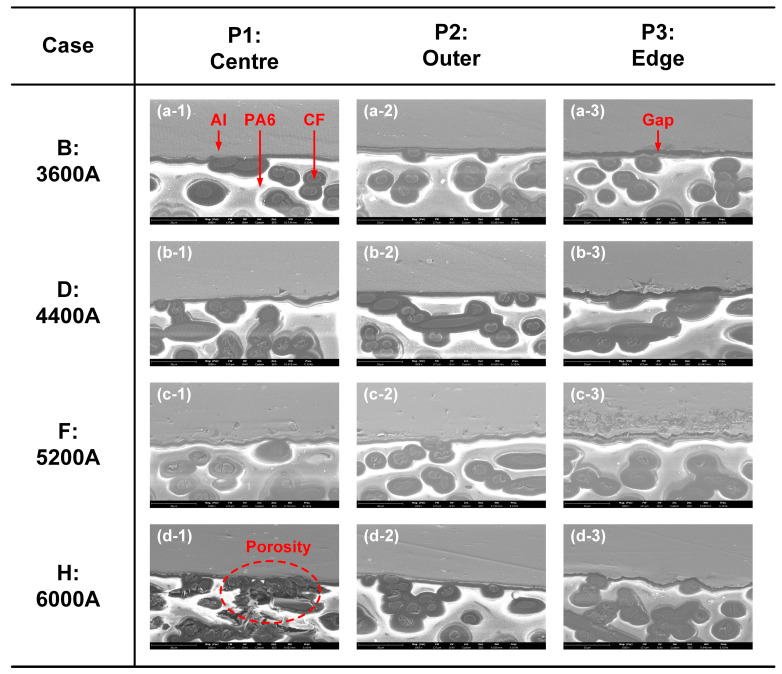
The SEM images of three typical positions at Al–CFRP interface in four welding conditions. (**a-1**) Case B: centre (**a-2**) Case B: outer (**a-3**) Case B: edge (**b-1**) Case D: centre (**b-2**) Case D: outer (**b-3**) Case D: edge (**c-1**) Case F: centre (**c-2**) Case F: outer (**c-3**) Case F: edge (**d-1**) Case H: centre (**d-2**) Case H: outer (**d-3**) Case H: edge.

The SEM images of three typical positions at the Al–CFRP interface in the four welding conditions are compared in Figure 5. The Al5052 sheet showed a grey colour, and the CFs were a dark colour. PA6 had poor conductivity; hence, the electrons were more likely to accumulate on the surface, which resulted in a bright appearance.

P1 was located at the centre of overlap zone, where the interface temperature was highest and the bonding between dissimilar materials was achieved. The Al and CFRP surfaces were tightly connected. However, the increasing welding current led to an excessive heat input and overheating of CFRP in Case H. The polymer matrix was decomposed significantly, as marked in Figure 5(d-1). In trying to achieve a strong bonding, this was a negative result.

In contrast, the interface temperature in P2 was relatively lower than in P1. Therefore, the interface bonding quality in all cases was good except for Case B. In Figure 5(a-2), the Al–CFRP interface was partly connected due to the low temperature and insufficient chemical reactions. The thermal degradation of CFRP was not observed at P2. P3 presented the edge of the joining zone, where the squeezed plastic formed a thin layer between the Al5052 and CFRP sheets. Therefore, the local interface temperature was lowest and there was always a clear gap between dissimilar materials. This indicated that the local joining strength should be the weakest in the entire joining zone.

**Figure 6:**
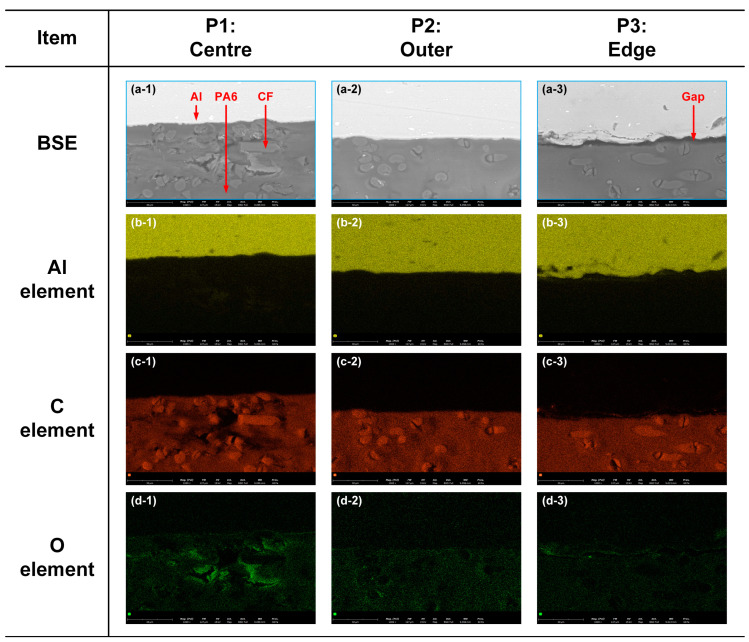
Distribution of the main elements in cross-sections of Case H: 6000 A. (**a-1**) BSE: centre (**a-2**) BSE: outer (**a-3**) BSE: edge (**b-1**) Al: centre (**b-2**) Al: outer (**b-3**) Al: edge (**c-1**) C: centre (**c-2**) C: outer (**c-3**) C: edge (**d-1**) O: centre (**d-2**) O: outer (**d-3**) O: edge.

In addition, EDS was performed for Case H to detect the main element distribution in three locations, as depicted in Figure 6. Figure 6a shows the backscattered-electron (BSE) images of the three locations on the Al–CFRP interface in Case H. The aluminium element only existed in the Al5052 sheet; therefore, it accumulated at the upper zone, as depicted in Figure 6b. As the main element of PA6, in Figure 6c, carbon is illustrated as red particles, which can be observed in the lower region. The CFs were rich in carbon elements; hence, the local signal intensity was relatively higher. The dark area in the CFRP in Figure 6(c-1) with low signal intensity indicated that the local polymer matrix had decomposed and formed a micro-hole. The distribution of oxygen is shown in Figure 6d. The oxygen signal was more significant in CFRP than in Al5052 since it is the main element of PA6. In particular, the O element was accumulated in the thermal degradation region of the plastic, as depicted in Figure 5(d-1), showing that oxygen in the atmosphere joined the thermal decomposition process of CFRP during welding.

### 3.2. Evaluation of Joining Strength

**Figure 7:**
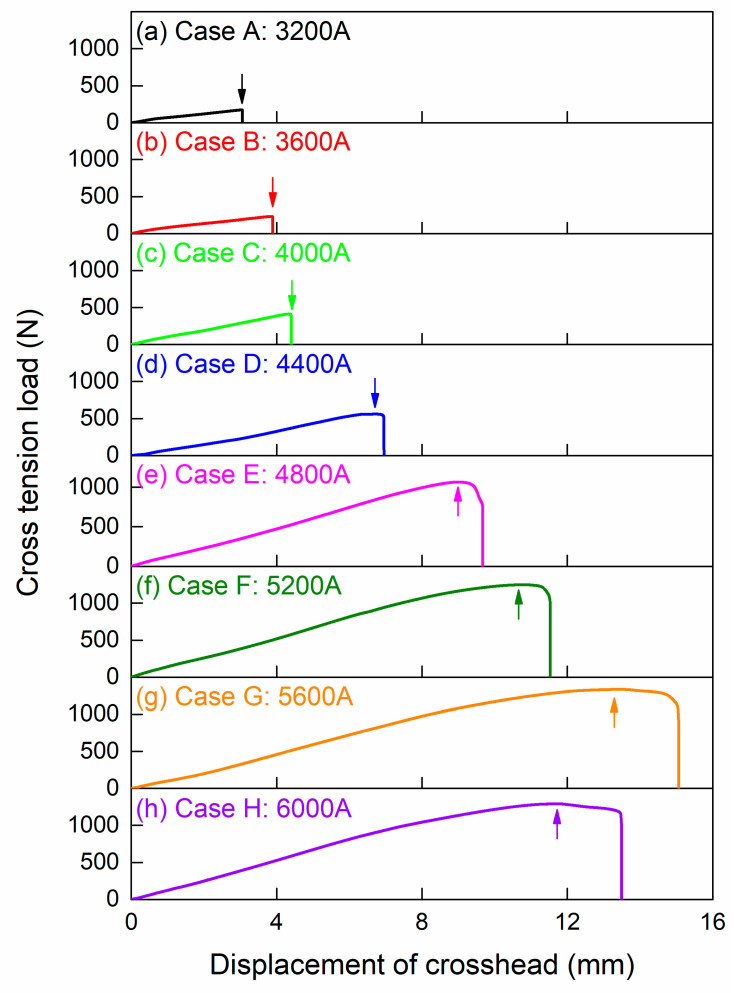
The results of cross-tension test with different welding conditions.

The cross-tension test results of different welding conditions are compared in Figure 7. With the continuous increase in welding current, the COS-RSW joints presented two fracture modes, whose transformation process could be divided into four conditions.

When the welding current was low, e.g., in Cases A, B and C, the load–displacement curves dropped immediately after the peaks. The COS-RSW joints endured a short crosshead displacement and presented a relatively low strength. In this condition, welded joints presented a brittle fracture. As depicted in Figure 7d, when the welding current increased to 4400 A in Case D, the larger heat input had a positive effect on joining strength. At this time, the load–displacement curve crossed its peak and then underwent a brief fracture process. Although the welded joint experienced a brittle-dominated failing process, the fracture mode changed into another one. 

In Cases E, F and G, there was a clear drop stage on the load–displacement curve after it crossed the peak—the joining zone in COS-RSW joints failed gradually; therefore, the fracture process was ductile-dominated. Both peak load and crosshead displacement were increased under a larger welding current. Finally, the welding current increased to 6000 A in Case H. The COS-RSW joint still presented a ductile fracture while its mechanical performance declined compared with Case G. This might have been induced by the thermal degradation of the polymer matrix near the Al–CFRP interface.

**Figure 8:**
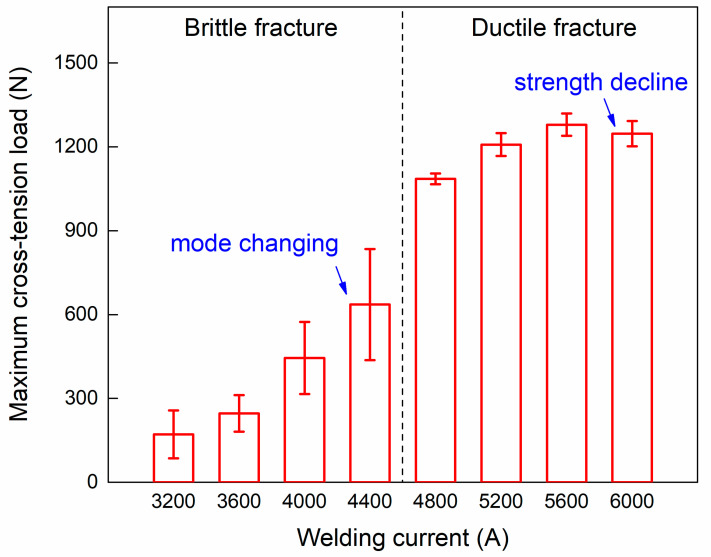
Influence of welding current on the joining strength of COS-RSW joints.

The joining strength of COS-RSW joints with different welding currents is summarized in Figure 8. The column and error bars represent the average and standard deviation of the load, which were calculated by three samples measurement in each case. When the welding current was lower than 4400 A, the welded joint presented a brittle fracture. The maximum cross-tension load increased with the higher welding current while the fluctuation in strength was significant. In Case D (4400 A), the maximum load was about 636 ± 198 N and the fracture mode started changing into ductile. When the welding current reached 4800 A, the fracture mode was ductile-dominated. The joining strength of the COS-RSW joint was almost double and the value was stable. This indicated that the ductile fracture could represent a strong bonding between Al and CFRP. The maximum load appeared under a 5600 A welding current, which was about 1279 ± 40 N. The mechanical performance of COS-RSW joints decreased clearly when the current reached 6000 A, which indicated that the overheating and thermal degradation of CFRP had a negative impact on the joining strength.

### 3.3. Analysis of Fracture Surface

**Figure 9:**
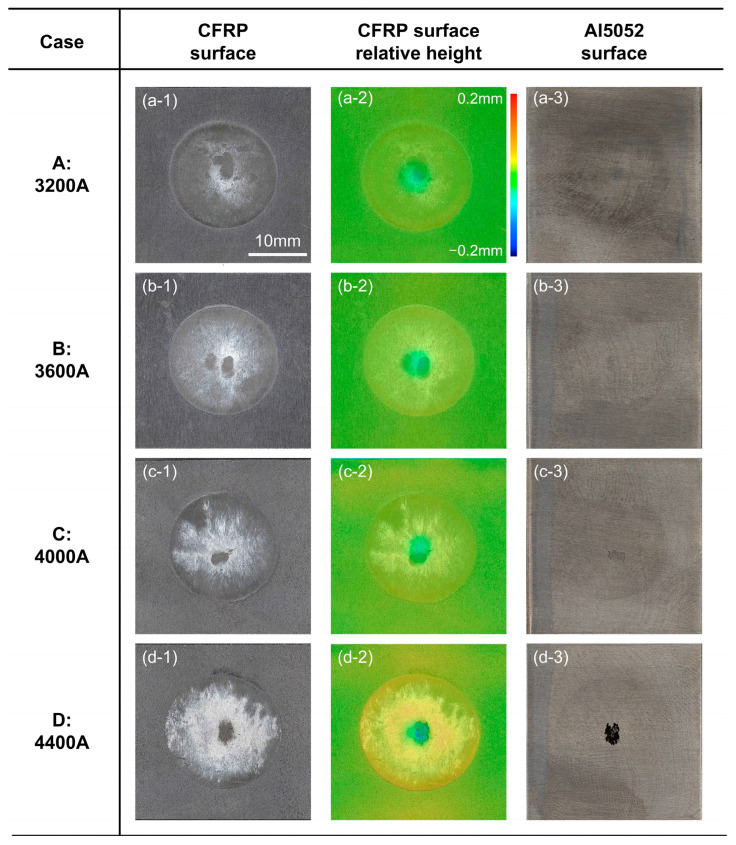
The macro-scale analysis of fracture surface in Cases A–D. (**a-1**) Case A: CFRP (**a-2**) Case A: relative height of CFRP (**a-3**) Case A: Al5052 (**b-1**) Case B: CFRP (**b-2**) Case B: relative height of CFRP (**b-3**) Case B: Al5052 (**c-1**) Case C: CFRP (**c-2**) Case C: relative height of CFRP (**c-3**) Case C: Al5052 (**d-1**) Case D: CFRP (**d-2**) Case D: relative height of CFRP (**d-3**) Case D: Al5052.

**Figure 10:**
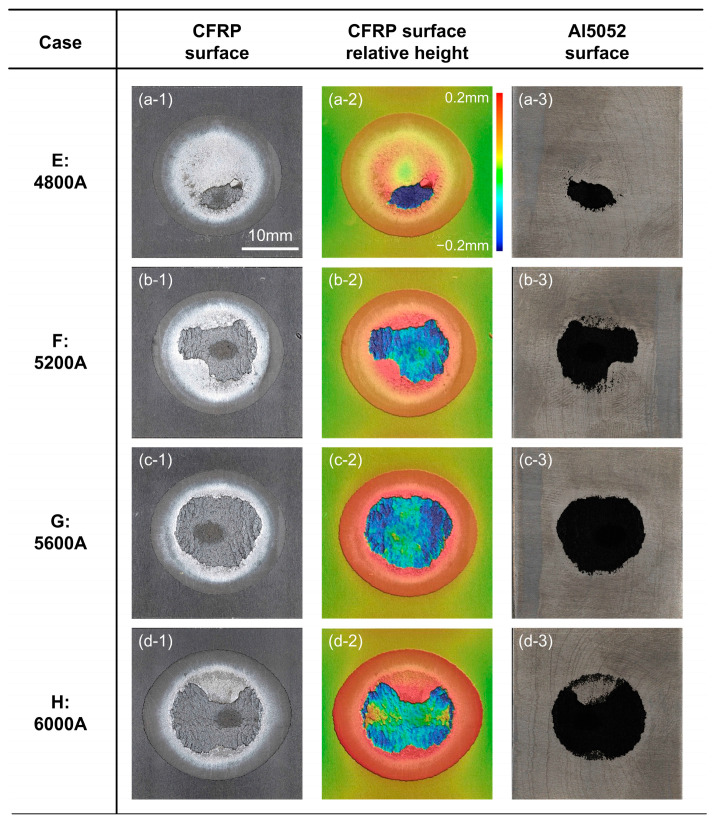
The macro-scale analysis of fracture surface in Cases E–H. (**a-1**) Case E: CFRP (**a-2**) Case E: relative height of CFRP (**a-3**) Case E: Al5052 (**b-1**) Case F: CFRP (**b-2**) Case F: relative height of CFRP (**b-3**) Case F: Al5052 (**c-1**) Case G: CFRP (**c-2**) Case G: relative height of CFRP (**c-3**) Case G: Al5052 (**d-1**) Case H: CFRP (**d-2**) Case H: relative height of CFRP (**d-3**) Case H: Al5052.

The macro-scale analysis of fracture surfaces, including the morphology and relative height measurement of CFRP, are summarized in Figure 9 and Figure 10.

In Figure 9a–c, one can see that the fracture surface morphology of Cases A, B and C was almost identical. There was a narrow region that showed a negative relative height in the centre of the overlap zone, which indicated that the melted plastic was squeezed out from the original location under the electrode force. On the contrary, the squeezed flowing plastic generated a circle area with positive relative height, which marked the boundary of the joining zone. The CFRP basement looked dark but some regions in the joining zone presented a light grey colour. This indicated that the plastic experienced a tensile deformation during the failing process, thereby appearing as a stress whitening phenomenon [23]. There was no residual CFRP on the Al5052 sheet, which showed that a fracture occurred on the Al5052–CFRP interface. The joining strength was relatively weak in such a condition; therefore, the corresponding fracture mode was brittle.

Case D had a larger welding current and heat input, which was beneficial to the chemical bond between the silane coupling agent film and the molten plastic. Therefore, Case D showed a larger strength in the cross-tension test and the stress whitening in the CFRP fracture surface was more apparent, as depicted in Figure 9(d-1). Significantly, one can observe the residual CFRP on Al sheet, as shown in Figure 9(d-3). This indicated that the local failure appeared in the polymer matrix rather than the Al–CFRP interface. Accordingly, the fracture mode of Case D was brittle-dominated but presented a tendency to alter into ductile failure.

With the increase in welding current and heat input in Cases E, F and G, more and more plastic was melted and squeezed out to form a circle layer, whose area and thickness became larger in the relative height measurement. At the same time, much of the CFRP was torn from polymer matrix and remained on the Al sheet, marked as a dark blue and negative relative height. This region also provided a strong bonding which was powerful enough to destroy the plastic. In Figure 10(b-2), one can see that the edge of blue area showed a clear red colour, which showed that the local plastic experienced an intense tensile deformation, but was still connected with the basement. On the outer region, the significant stress whitening indicated that the local fracture occurred along the interface. At the edge of the joining zone, its morphology was similar to the CFRP basement; hence its contribution to joining should have been limited. Therefore, the joining zone of Cases E, F and G failed stage-by-stage along the outside-in direction, which explained the ductile fracture mode in the cross-tension test.

The welding current and heat input in Case H was the highest. Therefore, the polymer matrix might have overheated and decomposed near the interface. In Figure 10(d-3), some Al surface was exposed and surrounded by plastic. This indicated that the bonded sheets were separated along the interface in this region. The area of torn CFRP decreased; therefore, the maximum cross-tension load was also reduced in Case H.

**Figure 11:**
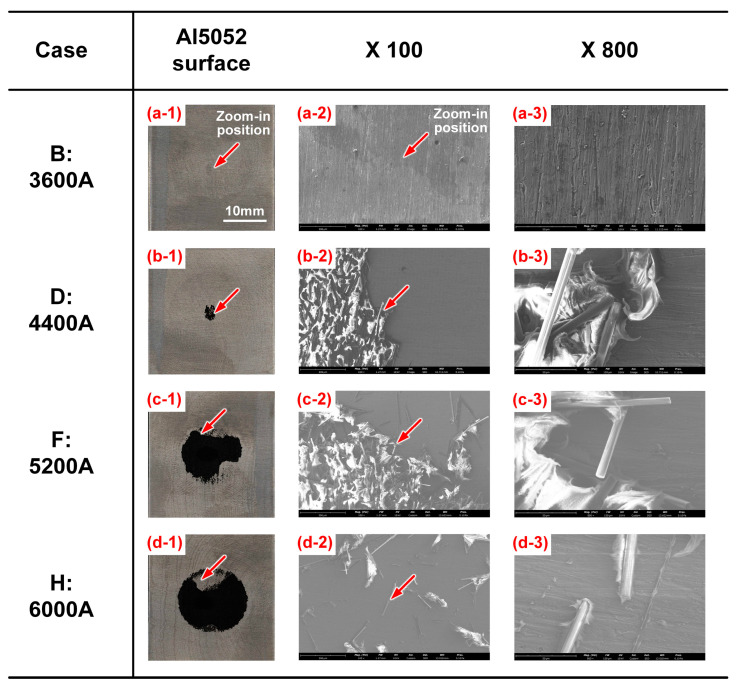
The SEM images of fracture surface on Al5052 sheet. (**a-1**) Case B: Al surface (**a-2**) Case B: X 100 (**a-3**) Case B: X 800 (**b-1**) Case D: Al surface (**b-2**) Case D: X 100 (**b-3**) Case D: X 800 (**c-1**) Case F: Al surface (**c-2**) Case F: X 100 (**c-3**) Case F: X 800 (**d-1**) Case H: Al surface (**d-2**) Case H: X 100 (**d-3**) Case H: X 800.

The SEM images of Al5052 surfaces in specific cases are compared in Figure 11. The positions marked by red arrows were magnified 100 times and 800 times to clarify the microstructure. As depicted in Figure 11a, the Al5052 surface was always clean; few residual polymer matrix and carbon fibres can be observed even under high magnification. This indicated that the failure appeared at the Al–CFRP interface; therefore, Case B presented a brittle fracture in the cross-tension test. In contrast, a little CFRP can be observed on Al sheet in Case D. The boundary between Al and the remaining CFRP is obvious in Figure 11(b-2), which shows that the local fracture was altered into plastics. This phenomenon was more significant in Case F due to higher heat input and sufficient chemical reaction. In Figure 11(c-3), the characteristics of PA6 showed that it experienced an intense tensile deformation before failure. The welded joints demonstrated a higher joining strength and ductile fracture mode. In Case H, the welding current and heat input were exceeded to trigger the thermal degradation of plastic, which had a negative impact on the local bonding quality. The connected Al and CFRP sheets were separated at the interface due to the weakened joining strength. It can be seen that the exposed Al surface was relatively clean, and a few carbon fibres were surrounded by residual PA6 with sporadic distribution, as shown in Figure 11(d-2),(d-3).

### 3.4. Subdivision of Joining Zone

As previously mentioned, the morphology of the fracture surface on the CFRP sheet was quite distinct in different positions, which indicated that the local plastic experienced unique failure processes. Thus, the joining zone could be divided into some subregions according to the morphological characteristics, whose contribution to joining strength should be specific.

**Figure 12:**
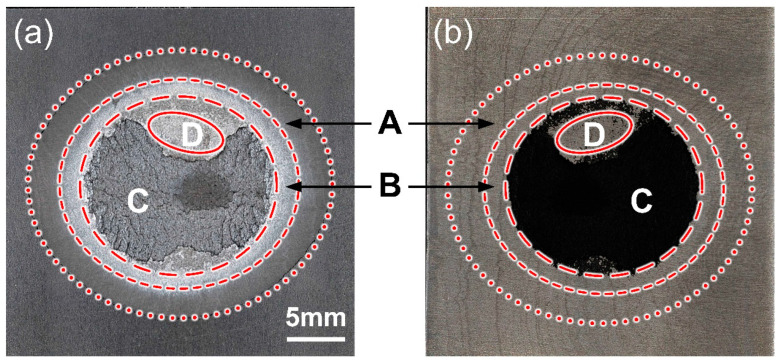
Subdivision of joining zone in (**a**) CFRP sheet and the relative regions in (**b**) Al5052 sheet.

In the present research, the fracture surface in Case H had the most abundant information and the joining zone could be subdivided into four parts, as depicted in Figure 12. Zone A was defined as the squeezed zone (SZ), which was generated by the squeezed flowing polymer matrix. The appearance of the SZ was similar to the CFRP basement and its contribution to the joining strength was limited. There was no residual plastic in the SZ on Al5052 sheet, which showed that the failure occurred at the interface. Zone B, marked by a half-dashed line, was defined as the adhesion zone (AZ). The Al5052 and CFRP were still separated along the interface in the AZ since the Al surface was relatively clean. However, the CFRP surface showed a light grey colour and stress whitening phenomenon, which indicated that the plastic experienced tensile deformation during fracture. Therefore, the bonding in the AZ was successful but not powerful enough.

Zone C, marked by a dashed line was defined as cohesion zone (CZ). Much of the CFRP was torn from the polymer matrix and remained on the Al5052 sheet, which indicated that the fracture occurred in the plastic rather than Al–CFRP interface. Due to the large welding current and sufficient interfacial chemical reaction, the bonding in the CZ should have been strongest in the entire joining zone, which gave a major contribution to the joining strength.

Zone D, marked by a solid line, was defined as the decomposed zone (DZ), where the CFRP was overheated by the excessive heat input. The polymer matrix started to decompose and the effective bonding area between dissimilar materials was reduced. Therefore, the local joining strength was too weak to tear the plastic. The fracture appeared on the interface and one can observe the exposed Al surface in residual CFRP on the Al5052 sheet.

**Figure 13:**
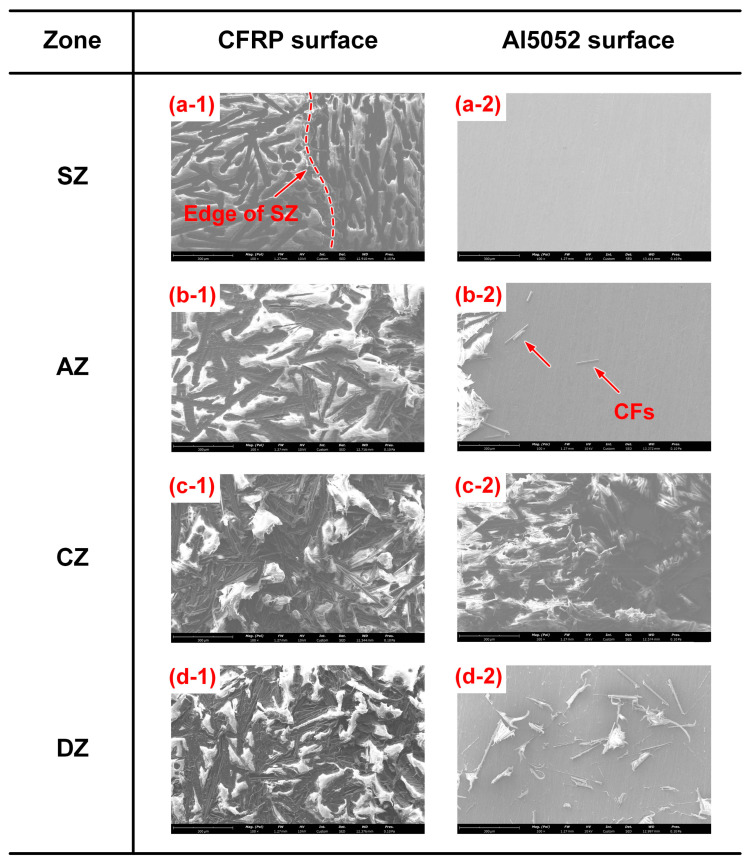
The SEM images of each subregion in CFRP and Al5052 sheets. (**a-1**) SZ on CFRP (**a-2**) SZ on Al5052 (**b-1**) AZ on CFRP (**b-2**) AZ on Al5052 (**c-1**) CZ on CFRP (**c-2**) CZ on Al5052 (**d-1**) DZ on CFRP (**d-2**) DZ on Al5052.

The SEM images of each subregion in the CFRP and Al5052 sheets are compared in Figure 13. Figure 13(a-1) shows the edge of the SZ; the appearance of the SZ (left) and CFRP basement (right) was almost identical. The smooth surface showed that the interfacial bonding in SZ was limited. The corresponding Al surface was relatively clean. As a comparison, the CFRP surface in the AZ looked rougher, which indicated the plastic endured a slight pulling before fracture. A few carbon fibres can be observed on the Al surface, as marked in Figure 13(b-2). The CZ provided the most robust bonding and much CFRP was torn from polymer matrix. The PA6 in the CFRP surface showed a significant characteristic of tensile deformation; the spatial orientation of carbon fibres was disordered. The surface of residual CFRP on the Al sheet was quite rough and the relatively lower zone was dark on the right of Figure 13(c-2). Figure 13d shows the fracture surface in the DZ. Due to the overheating and thermal degradation of polymer matrix, numerous CFs were exposed on the CFRP sheet. Meanwhile, the CFs were distributed flatly on the CFRP surface and some residual plastics could be observed on the Al surface, which indicated that the local fracture in DZ appeared on Al–CFRP interface.

### 3.5. XPS Result of the Subregions in Joining Zone

**Figure 14:**
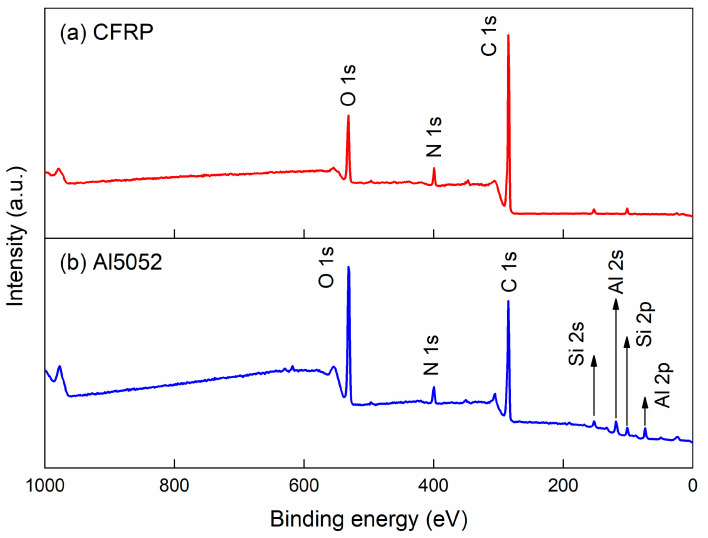
The low-resolution (survey) XPS spectra of (**a**) CFRP sheet and (**b**) Al5052 sheet.

The low-resolution (survey) XPS spectra collected from the CFRP and Al5052 sheets are depicted in Figure 14. The polymer matrix of CFRP in the present research was PA6, which has a chain structure of [NH(CH_2_)_5_CO]_n_. Therefore, the major signal peaks represented the O, N and C elements. The principal constituent of silane coupling agent was C_3_H_6_NHC_2_H_4_NH_2_Si(OCH_3_)_3_. The surface treatment of the Al5052 sheet can introduce some polysiloxane layers to the Al surface, which could be inter-connected via a loose network structure [24]. Hence, one could also find the signal peaks of Al and Si elements on the Al5052 sheet.

**Figure 15:**
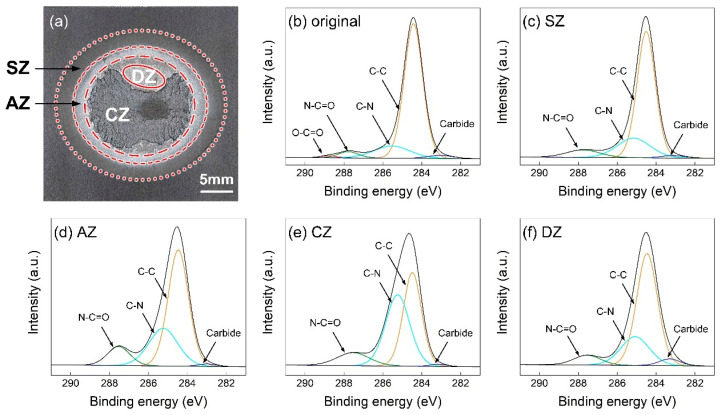
The high-resolution C1s XPS region spectra on CFRP sheet. (**a**) subregion of joining zone (**b**) C1s spectra of original CFRP (**c**) C1s spectra of SZ (**d**) C1s spectra of AZ (**e**) C1s spectra of CZ (**f**) C1s spectra of DZ.

The high-resolution C1s XPS region spectra in the subregion of joining zone on CFRP sheet are compared in Figure 15. The information on the original CFRP surface is given in Figure 15b. The highest and lowest signal peaks represented the C-C bond and carbide, respectively. As the primary bonds in the PA6 unit, the C-N peak appeared at about 285.5 eV, and the N-C=O peak occurred at about 287.8 eV [25]. In addition, the peak located at about 288.8 eV indicated the O-C=O bond [26], which was the end group on the PA6 chain, and remained, indistinctly, on the CFRP surface.

The strong bonding between the CFRP and the silane coupling agent was based on the chemical reaction. The carboxyl group (-COOH) on the end of PA6 chain and the amide group (-NH_2_) on the silane coupling agent can generate an N-C=O bond via the amidation process. In the SZ and AZ, the local plastic was melted during welding to trigger the chemical reaction. Therefore, the O-C=O peak disappeared while the signal intensity of N-C=O was increased, as depicted in Figure 15c,d. In particular, the AZ experienced a more sufficient heating process and the chemical reaction was more adequate, which reflected a significant peak of N-C=O and a better macro strength.

The cohesive zone (CZ) had a powerful interfacial bonding strength and the fracture occurred in the CFRP. At this moment, the polymer matrix experienced a significant plastic deformation. The well-entangled chains of polymer were separated and exposed on the surface, which resulted in a stronger signal of the C-N bond, as shown in Figure 15e. For the decomposition zone (DZ), the plastic started to degrade and local fracture returned to the interface. Hence, the signal intensity of C-N bond decreased while the peak of the carbide increased significantly.

**Figure 16:**
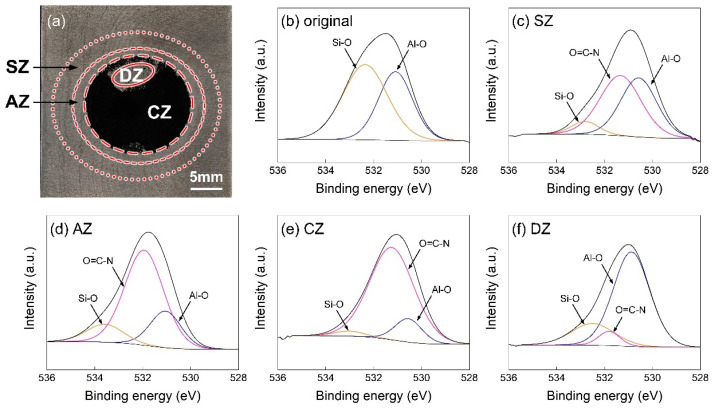
The high-resolution O1s XPS region spectra on Al5052 sheet. (**a**) subregion of joining zone (**b**) O1s spectra of original Al5052 (**c**) O1s spectra of SZ (**d**) O1s spectra of AZ (**e**) O1s spectra of CZ (**f**) O1s spectra of DZ.

Figure 16 shows the high-resolution O1s XPS region spectra in the subregion of the joining zone on the Al5052 sheet. In Figure 16b, two peaks could be distinguished at about 531.0 eV and 532.9 eV, which indicated the Al-O and Si-O bonds [27] on the pretreated Al surface, respectively. In the SZ and AZ, the molten PA6 reacted with silane coupling agent; therefore, the O=C-N bond at about 531.6 eV could be detected on the Al surface [28]. The signal intensity of O=C-N was stronger in the AZ than SZ, which was induced by a higher interface temperature and the related adequate reaction. In particular, Figure 16e shows the CZ posited in the narrow region between the AZ and DZ. Some plastic remained on the Al sheet; hence, the signal intensities of Al-O and Si-O bonds were weak. As depicted in Figure 16f, the CFRP started to decompose in the DZ and only a little polymer matrix remained on the Al surface. Therefore, the signal intensity of O=C-N was decreased significantly.

### 3.6. Fracture Behaviours and Mechanism

In the current research, the COS-RSW joints presented brittle fractures and ductile fractures in the cross-tension test, which included two sub-modes. The fracture behaviours and corresponding mechanisms are discussed in detail.

**Figure 17:**
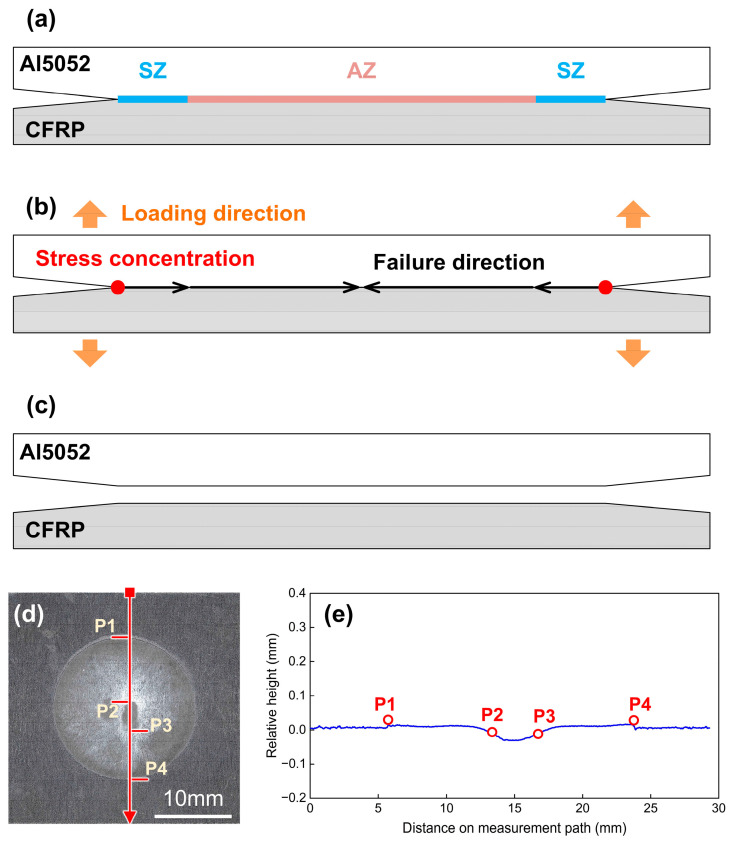
The mechanism of brittle fracture in Case B: 3600 A. (**a**) joining zone components (**b**) schematic of failure direction (**c**) schematic of fracture feature at interface (**d**) actual fracture surface on CFRP (**e**) relative height distribution.

Figure 17 illustrates the fracture behaviours and mechanism in Case B with a 3600 A welding current. Since the welding current and heat input were relatively low, the chemical reaction on the Al–CFRP interface was insufficient, which resulted in a weak bonding and the joining zone included the SZ and AZ, as shown in Figure 17a. During the cross-tension test, the stress concentration appeared at the edge of the joining zone, and failure occurred along the interface. Finally, the Al5052 surface was clean and the joints presented a brittle fracture. Figure 17d,e show the relative height distribution on the CFRP sheet in Case B. P1 and P4 mark the edge of joining zone but the boundary between the SZ and the AZ was unclear. The narrow drop zone between P3 and P4 indicated that the melted plastic was squeezed out from its original position.

**Figure 18:**
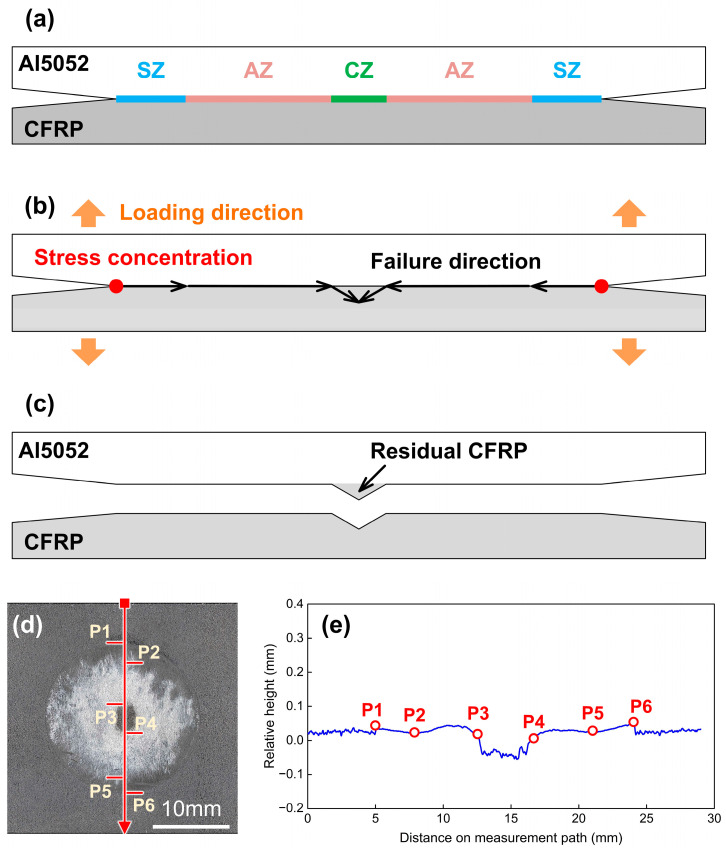
The mechanism of brittle-transition fracture in Case D: 4400 A. (**a**) joining zone components (**b**) schematic of failure direction (**c**) schematic of fracture feature at interface (**d**) actual fracture surface on CFRP (**e**) relative height distribution.

The fracture behaviour and mechanism of Case D is explained in Figure 18, which shows a brittle-transition fracture. Although the failure process was brittle-dominated, it showed the tendency to alter into a ductile one. The CZ appeared in the joining zone due to a larger welding current and better interface bonding. The failure occurred first along the interface in SZ and AZ, then moved into the CFRP from the edge of the CZ. Finally, a small amount of CFRP was torn from polymer matrix and remained on the Al5052 sheet, as illustrated in Figure 9(d-3) and Figure 18c. In the relative height measurement result, P1 and P6 mark the edge of joining zone. P2 and P5 show the boundary between the SZ and AZ. P3 and P4 mark the CZ where the CFRP was torn off due to the strong bonding.

**Figure 19:**
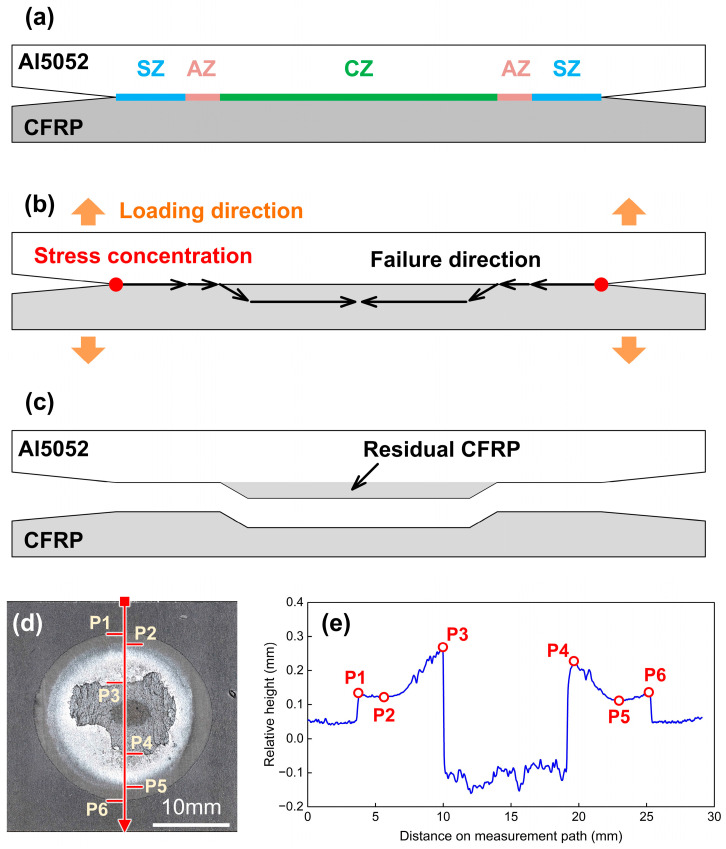
The mechanism of ductile fracture in Case F: 5200 A. (**a**) joining zone components (**b**) schematic of failure direction (**c**) schematic of fracture feature at interface (**d**) actual fracture surface on CFRP (**e**) relative height distribution.

Figure 19 shows the fracture characteristics in Case F. When the welding current increased continuously, the area of the CZ expanded significantly with the higher heat input. At the same time, the AZ became smaller, as depicted in Figure 19a. In this condition, the failure path would move into the CFRP sheet early. The destruction of plastic required a certain tensile deformation and external load. Therefore, the COS-RSW joint presented a ductile fracture mode and a sizeable joining strength. A large amount of the CFRP was torn from polymer matrix and remained on the Al sheet, as illustrated in Figure 10(f-3) and Figure 19c. In the relative height measurement result, P1 and P6 show the outside of the SZ, the boundary between SZ and AZ is distinguished by P2 and P5. P3 and P4 mark the edge of CZ. In the path of P2–P3 and P5–P4, the relative height in AZ was increased obviously on the outside-in direction, where the surface also became rougher. this showed that the subregions in AZ experienced different tensile deformations, which were induced by the different interface temperatures and local bonding strengths.

**Figure 20:**
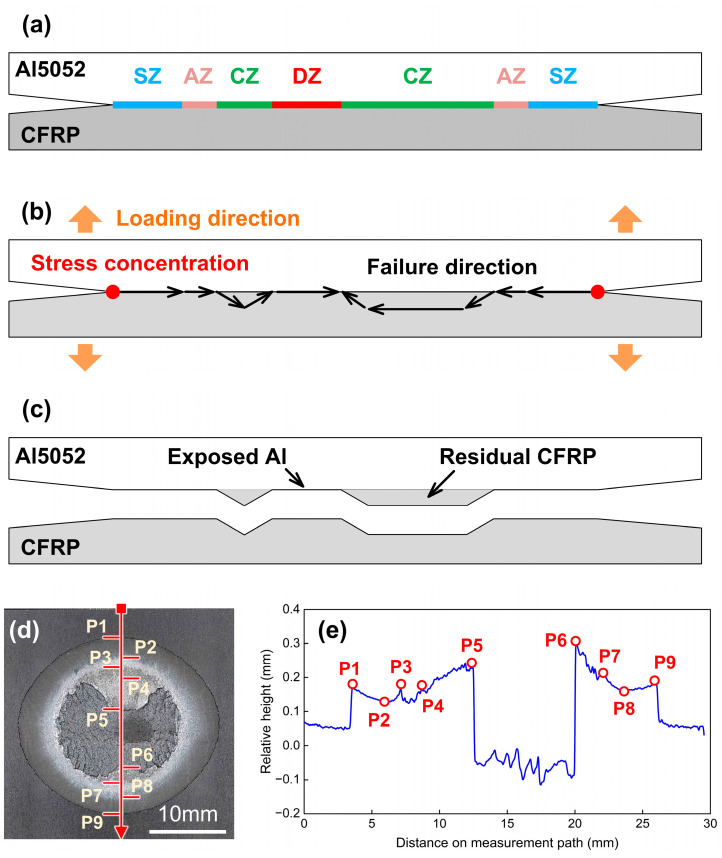
The mechanism of ductile-degradation fracture in Case H: 6000 A. (**a**) joining zone components (**b**) schematic of failure direction (**c**) schematic of fracture feature at interface (**d**) actual fracture surface on CFRP (**e**) relative height distribution.

When the welding current increased to 6000 A in Case H, the COS-RSW joint presented a ductile-degradation fracture behaviour, whose mechanical performance was decreased. The DZ appeared in the joining zone in which the interface temperature can exceed the decomposition temperature of CFRP significantly, thereby leading to the thermal degradation of the polymer matrix. The local joining strength in the DZ was weakened and insufficient to tear the CFRP. Therefore, the failure path would return to the Al–CFRP interface rather than pass through the plastic, as illustrated in Figure 20b. Finally, one can see the Al surface exposed in the residual CFRP, as shown in Figure 10(h-3) and Figure 20c. The relative height distribution in Case H was similar to that in Case F. In Figure 20e, P5 and P7 marked the edge of large CZ. The significant drop between P5 and P6 indicated that the CFRP was torn from polymer matrix. The rough surface between P6 and P7 showed that the local fracture also occurred in the plastics, which can be proved by the residual CFRP distributions in Figure 10(h-3). On the left, a narrow drop between P3 and P4 showed the CZ which was located on the other side of joining zone. Between two CZ, P4 and P5 marked the boundary of DZ, where the CFRP surface was raised but not torn from basement. It also indicated the decline in local bonding strength.

## 4. Conclusions

In the present research, coaxial one-side resistance spot welding was used to join Al5052 and CFRP sheet. The mechanical performance of the cross-lap joint was clarified experimentally. The cross-section of the welded joint and the fracture surfaces was subjected to multi-scale characterization. The joining zone was subdivided according to the morphology and analysed via XPS. The fracture behaviour and mechanism of cross-lap joints are also discussed in detail. The following conclusions can be drawn:

1. The maximum cross-tension load of COS-RSW cross-lap joints reached about 1279 ± 40 N under 5600 A welding current. A larger welding current had a negative impact on the joining strength due to the overheating and thermal degradation of CFRP.
2. In Case H, with a 6000 A welding current, the thermal degradation of CFRP could be observed on the cross-section. The O element was enriched in the decomposed area, which indicated that oxygen in the atmosphere joined the thermal degradation process of CFRP during welding.
3. The joining zone of COS-RSW cross-lap joints could be divided into four subregions according to the morphology, which, from outside to inside, were the squeezed zone, adhesion zone, cohesion zone and decomposed zone. Local fracture appeared in the CFRP matrix in the cohesion zone, while the joints failed at the Al–CFRP interface in the squeezed zone, adhesion zone and decomposed zone due to insufficient or exceeded heat input.
4. After welding, O-C=O bonds disappeared on the CFRP surface while the O=C-N bond was detected on the Al5052 surface. The chemical reaction between PA6 and the silane coupling agent provided the joining force between dissimilar materials.
5. The COS-RSW cross-lap joints presented brittle and ductile fracture behaviours in the cross-tension test, which included two sub-modes—the brittle-transition mode and ductile-degradation mode. The alternation of fracture characteristics was induced by heat input and the corresponding joining zone components.
6. In order to improve the mechanical performance of the COS-RSW joint, it was suggested to optimize welding parameters and control interface temperature to maintain sufficient interfacial reaction but avoid the thermal degradation of CFRP.

## Figures and Tables

**Table 1:** COS-RSW process parameters.

Case	Welding Current (A)	Welding Time (s)	Electrode Force (N)	Holding Time (s)
A	3200	0.45	2450	10
B	3600	0.45	2450	10
C	4000	0.45	2450	10
D	4400	0.45	2450	10
E	4800	0.45	2450	10
F	5200	0.45	2450	10
G	5600	0.45	2450	10
H	6000	0.45	2450	10

## Data Availability

The data used to support the findings of this study are included within the article.
